# EPMOSt: An Energy-Efficient Passive Monitoring System for Wireless Sensor Networks

**DOI:** 10.3390/s140610804

**Published:** 2014-06-19

**Authors:** Fernando P. Garcia, Rossana M. C. Andrade, Carina T. Oliveira, José Neuman de Souza

**Affiliations:** 1 Federal Institute of Education, Science and Technology (IFCE), CEP 60040-215, Fortaleza, Ceará, Brazil; 2 Group of Computer Networks, Software Engineering and Systems (GREat), Federal University of Ceará, CEP 60455-760, Fortaleza, Ceará, Brazil; E-Mails: rossana@ufc.br (R.M.C.A.); carina@great.ufc.br (C.T.O.); neuman@ufc.br (J.N.S.)

**Keywords:** passive monitoring, energy-efficient, wireless sensor networks

## Abstract

Monitoring systems are important for debugging and analyzing Wireless Sensor Networks (WSN). In passive monitoring, a monitoring network needs to be deployed in addition to the network to be monitored, named the target network. The monitoring network captures and analyzes packets transmitted by the target network. An energy-efficient passive monitoring system is necessary when we need to monitor a WSN in a real scenario because the lifetime of the monitoring network is extended and, consequently, the target network benefits from the monitoring for a longer time. In this work, we have identified, analyzed and compared the main passive monitoring systems proposed for WSN. During our research, we did not identify any passive monitoring system for WSN that aims to reduce the energy consumption of the monitoring network. Therefore, we propose an Energy-efficient Passive MOnitoring SysTem for WSN named EPMOSt that provides monitoring information using a Simple Network Management Protocol (SNMP) agent. Thus, any management tool that supports the SNMP protocol can be integrated with this monitoring system. Experiments with real sensors were performed in several scenarios. The results obtained show the energy efficiency of the proposed monitoring system and the viability of using it to monitor WSN in real scenarios.

## 1. Introduction

The miniaturization of electronic components and the evolution of wireless communication technologies have stimulated the development and use of Wireless Sensor Networks (WSNs) in various applications, such as environmental monitoring, seismic detection, structural health monitoring, and smart spaces, among others. In general, WSNs consist of small sensors which use wireless short range communication. Furthermore, these networks have severe energy consumption, processing power, memory, and bandwidth constraints [1].

Monitoring is important for debugging and analyzing the operation of WSNs. By using a monitoring system, several pieces of information about the operation of the network can be obtained, such as topology discovery, reboot of nodes, isolated nodes, routing loops, packet loss, and network latency, among others [2].

In a WSN, network monitoring can be divided into active monitoring and passive monitoring. In active monitoring, code lines are inserted in the application running in sensor nodes to obtain information about the operation of the network. In this case, the monitoring packets are sent along with data packets of the network, modifying the behavior and operation of the monitored network and consuming the resources of this network. For example, in the active monitoring system Simpathy [3] about 30% of the bandwidth is used by monitoring traffic. Furthermore, faults in the network may affect the monitoring system and prevent the delivery of monitoring information when it is most needed.

In passive monitoring, a monitoring network needs to be deployed in addition to the network to be monitored, named the target network. The monitoring network captures and analyzes packets transmitted by the target network, not consuming any resources of the target network. Furthermore, a fault in the target network does not affect the operation of the monitoring system. However, the deployment of an additional network with the purpose of monitoring the target network increases the overall cost of the WSN.

Therefore, passive monitoring systems are more suitable when the goal is to reduce the use of target network resources and/or isolate the faults of the target network from those of the monitoring network. In this paper, we focus on passive monitoring.

The lifetime of a WSN can be up to several years and not all problems arise during the first weeks after the deployment of the network [4]. Therefore, an energy-efficient monitoring system is necessary when there is a need for monitoring a WSN in a real scenario over long periods because the lifetime of the monitoring network is extended and, consequently, the target network benefits from the monitoring for a longer time. Liu *et al.* [5] describe the use of a WSN for monitoring the oceans and emphasize the importance of monitoring this network using an energy-efficient passive monitoring system.

All things considered, initially, we have identified, analyzed and compared the main passive monitoring systems proposed for WSN. During our research, we did not identify any passive monitoring system for WSN that aims to reduce the energy consumption of the monitoring network. Therefore, in this paper we propose an energy-efficient passive monitoring system for WSN named **E**nergy-efficient **P**assive **MO**nitoring **S**ys**t**em (EPMOSt). The main goal of this system is to reduce the energy consumption and, consequently, extend the lifetime of the monitoring network. Furthermore, EPMOSt provides monitoring information using an SNMP agent. In this way, any management tool that supports the SNMP protocol can be integrated with this monitoring system.

The remainder of the paper is organized as follows: in Section 2, the main passive monitoring systems proposed for WSN are analyzed and compared. Section 3 presents the monitoring system proposed in this paper and describes the details of its implementation. Section 4 describes the experiments and metrics used to evaluate the proposed monitoring system. Section 5 shows and discusses the results obtained from these experiments. Finally, Section 6 summarizes our contributions and identifies avenues for future work.

## 2. Passive Monitoring in WSNs

This section discusses the main passive monitoring systems proposed specifically for wireless sensor networks. In Sensor Network Troubleshooting Suite (SNTS) [6], nodes of the monitoring network (sniffers) listen passively on the communication channel and capture packets sent by the nodes of the target network. When capturing a packet, a sniffer includes a record in its non-volatile memory (e.g., flash memory) with the contents of the packet and a timestamp. After the period of data capture, sniffers are manually collected and records of the captured packets are transferred to a computer, where they are analyzed. The information obtained from the captured packets is displayed in a tool developed by the authors. The aim of SNTS is to help the developer of applications for WSN to find and fix faults during development time. However, it is not feasible to use the SNTS to monitor WSN deployed in real scenarios where it is impractical to collect the sniffers, for example in military applications or environmental monitoring applications.

In Sensor Network Inspection Framework (SNIF) [7], each sniffer has two radio interfaces, one of which is used to capture packets sent by the nodes of the target network and the other to send the captured packets to the sink node (e.g., a computer) through the monitoring network. The packets captured by sniffers are tagged with a timestamp and sent to the sink node, where duplicate packets are removed. Thus, the packets are analyzed and the information obtained from the monitoring is displayed in a tool developed by the authors.

In Pimoto [8], the target network is subdivided into monitoring islands. A sniffer is deployed in each monitoring island. The sniffer is responsible for capturing in promiscuous mode the packets sent by the nodes of the target network that are within its island and sending theses packets to a gateway (computer) using a Bluetooth radio. A gateway can receive the packets captured by several sniffers. The gateway includes the timestamp and the sniffer address in each captured packet and then it sends the packets to a server. The server analyzes and displays the captured packets with the well-known traffic analysis tool Wireshark [9] using a plugin developed by the authors. In Pimoto, captured packets can be viewed in Wireshark, but the analysis of packets must be performed by the network administrator. Furthermore, the use of Pimoto may be impractical to monitor a WSN that has too many nodes distributed in a wide geographic area because it requires an infrastructure composed of multiple gateways interconnected to the server.

In LiveNet [10], packets captured by sniffers can be stored in a flash memory or sent to a computer via a serial port for future analysis. On the computer, the captured packets are analyzed to obtain information about the behavior of the target network. Like SNTS, it is not feasible to use LiveNet to monitor WSN deployed in scenarios where it is impractical to collect the data stored in the flash memory of sniffers or to send the data collected by sniffers using a wired network, for example in military applications or environmental monitoring applications.

In Passive Monitoring System in Wireless (PMSW) sensor networks [11], each sniffer captures data packets and acknowledgment packets (ACKs) sent by the nodes of the target network that are within its coverage area, and sends the captured packets to its gateway (computer). A sniffer connects to only one gateway via a radio channel with frequency different from the frequency of operation of the target network. When the gateway receives the packets captured by its sniffers, it creates a local trace. Each record of the local trace contains the information of a packet and a timestamp based on the gateway's clock. Hence, each gateway sends the local trace to a server through a TCP/IP network. The server receives the traces sent by all gateways, and generates a global trace with duplicated records removed. Then the global trace is analyzed in order to detect faults and to assess the performance of the target network. The information obtained from this analysis is displayed in a tool developed by the authors. In PMSW, only data and ACK packets are captured, while control packets, such as routing packets and packets of cluster election, are not captured or analyzed.

Table 1 shows the comparative analysis of the monitoring systems described in this section. We analyze the following characteristics:

- Energy-efficient—indicates whether the system is concerned with minimizing the energy consumption of nodes of the monitoring network;
- Analysis mode—indicates whether the analysis of captured packets is performed online or offline;
- Captured packets – types of packets (data and/or control) captured by the monitoring network;
- Event analysis—indicates whether the monitoring system analyzes the captured packets to obtain information about the operation of the target network;
- Redundant packets—verifies whether the monitoring system captures redundant packets. Redundant packets are captured when two or more sniffers capture packets sent by the same node of the target network;
- Aggregation—verifies whether the monitoring system uses any mechanism to aggregate information of packets captured from the target network in order to reduce the number of packets transmitted by the monitoring network and, consequently, reduce the energy consumption of this network; and
- Visualization tool—describes the computational tool used to display information obtained from passive monitoring.

The characteristic *analysis mode* and *event analysis* were defined in [11], while the remaining characteristics were identified from the study of passive monitoring systems discussed in this section. The capture of these characteristics is an additional contribution of this work. The main conclusions obtained by observing Table 1 are:

- None of these systems is energy-efficient; in other words, the proposed solutions are not concerned with minimizing the energy consumption of nodes of the monitoring network. Energy efficiency is important when it is necessary to monitor sensor networks deployed in remote locations, where it is impractical to replace the batteries of sniffers;
- In SNIF, Pimoto and PMSW the analysis of captured packets is online. In SNTS and LiveNet captured packets are stored in non-volatile memory, which must be collected manually for future analysis (offline);
- SNTS, SNIF and PMSW analyze the captured data in order to detect fault events and/or assess the performance of the target network, while Pimoto and LiveNet only display the traces of captured packets;
- Only Pimoto does not capture redundant packets, because the target network is subdivided into monitoring islands and each island has only one sniffer. The capture of redundant packets increases the energy consumption of the monitoring network because sniffers transmit redundant packets through this network;
- None of these systems implements an aggregation mechanism of packets captured from a target network. Aggregation reduces the number of bytes transmitted by the monitoring network and hence reduces the energy consumption of this network; and
- Only Pimoto displays the monitoring information in a network management tool used by the community (*i.e.*, Wireshark), and none of these systems provides the monitoring information using an SNMP agent.

The monitoring systems compared in Table 1 have two properties: (1) they use an independent monitoring network consisting of several sniffers to capture in promiscuous mode the packets sent by the target nodes; (2) they do not perform any modification to the application executed by the target nodes. The operation of the monitoring systems which have both properties is independent of the target network. In this paper, we focus on these systems.

Others passive monitoring systems for WSN are proposed in [4,5,12–15], but these systems do not have the two properties mentioned above. The monitoring system proposed by Hanninen *et al.* [4] does not have the property (1) because it uses only one sniffer, which should be deployed close to the target nodes to be monitored. Sommer and Kusy [12] propose Minerva, a testbed architecture for distributed debugging of WSN. Minerva does not have the property (1) because each target node is physically connected, via its pin debug, to a sniffer. In Jiangwu *et al.* [13], the target network faults are detected by analyzing only the data received by the sink node. Thus, this system also does not have the property (1) because it does not use any monitoring network. The monitoring systems proposed by Liu *et al.* [5] and Yang *et al.* [14] do not have the property (2) because the application that is executed by the target node is modified to include the node address (*id*) in all packets which are routed through it. The system proposed by Romer and Ringwald [15] also does not have the property (2) because the application that is executed by the target node is modified to send data on the status of the node.

## 3. EPMOSt

As mentioned in Section 1, an energy-efficient passive monitoring system is necessary when we need to monitor a WSN in a real scenario for a long time; otherwise, the lifetime of the monitoring network can be much shorter than the lifetime of the target network, due to misuse of the energy of sniffers nodes. Thus, we propose an energy-efficient passive monitoring system for WSN, named EPMOSt, which reduces the energy consumption of the monitoring network. Furthermore, the proposed system provides monitoring information using an SNMP (Simple Network Management Protocol) agent. The SNMP agent allows the integration of the proposed system with any management tool with SNMP support, such as Nagios [16], Net-SNMP [17], SNMP MIB Browser Android Tool [18] and Manage Engine MIB Browser Free Tool [19].

**Figure 1.**
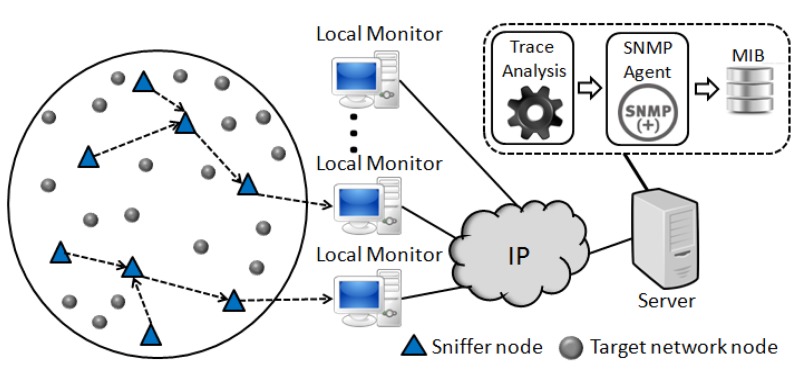
Overview of the EPMOSt.

Figure 1 shows the overview of the monitoring system EPMOSt, in which a monitoring network is deployed together with the target network. A monitoring network node (sniffer) captures, in a promiscuous mode, the packets sent by one or more nodes of the target network, inserts a timestamp for each captured packet, and aggregates the headers of several packets in a monitoring message. Then the sniffer sends this message, using the monitoring network, to the local monitor. The local monitor receives the monitoring messages from multiple sniffers and inserts the information of captured packets in a trace file (database) located on the server. The server analyzes the trace generated by one or more local monitors and obtains several pieces of information about the target network (*i.e.*, time at which each node awakens, packet loss, reboot of nodes, number of sent and received packets by each node, *etc.*). This information is available to the network administrator and it is also stored in a Management Information Base (MIB) to be accessed by an SNMP agent.

**Figure 2.**
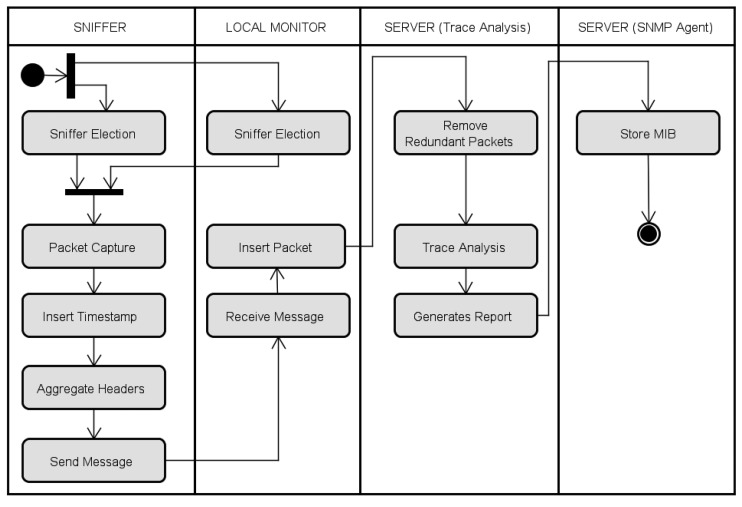
UML activity diagram of the EPMOSt.

Figure 2 shows the Unified Modeling Language (UML) activity diagram of the EPMOSt. In this system, in general the packets of a given node of the target network are captured by only one sniffer in order to reduce the transmission of redundant packets and hence reduce the energy consumption of the monitoring network.

The EPMOSt initially runs a mechanism (*Sniffer Election*) to elect the nodes of the target network that will have their packets captured by each sniffer. This election mechanism is performed by the sniffers and by the local monitor, taking into account the Received Signal Strength Indicator (RSSI). This mechanism is explained in detail in Section 3.1.1.

When capturing a packet of the target network, the sniffer inserts a timestamp in this packet. After capturing some packets, the sniffer may aggregate the headers of these packets in a monitoring message to send to the local monitor. The aggregation of headers aims to reduce the number of bytes sent through the monitoring network and, consequently, reduce the energy consumption of this network. In this case, only the information present in the headers of the packets sent by the target network is monitored. However, when there is a need to monitor the data (payload) sent by the target network, the system should not use this aggregation module. The aggregation mechanism is explained in detail in Section 3.1.2.

The local monitor receives the monitoring messages sent by sniffers and inserts the information of captured packets in a trace file (database) located on the server. The local monitor communicates with the server through an IP network. In some scenarios, it is necessary to deploy local monitors in different parts of the network due to the limited radio range of sniffers. In a more restricted scenario, only one local monitor can be used. In this case, the same computer can act as local monitor and server.

The main function of the server is to extract information about the target network from the trace generated by local monitors. For this purpose, initially, the server removes redundant packets that were inserted into the trace. Note that there may be redundant packets in the trace when two or more sniffers capture packets from the same sensor node of the target network. The algorithm to remove redundant packets is explained in detail in Section 3.3.

After this, the trace is analyzed to obtain information about the target network. The information obtained is used to generate a report to display for the network administrator. In particular, it is stored in an MIB by an SNMP agent. Therefore, any management tool based on SNMP protocol can communicate with the SNMP agent and display the information obtained from the monitoring of the target network. The implementation details of the system are presented in the following subsections.

### 3.1. Sniffer

In our implementation, the monitoring network uses, as sniffers, sensor nodes of the MicaZ platform, developed by Crossbow Technology. This platform was chosen because it is widely used in applications of WSN in general and it is used in related work in WSN [20,21] of the research group to which this work is linked. The monitoring application embedded in sniffers was developed using the programming language nesC (network embedded system C) [22] and it runs on the TinyOS [23] operating system.

#### 3.1.1. Sniffer Election

After the deployment of the monitoring network, sniffers and a local monitor start the *Sniffer Election* in order to elect the nodes of the target network that will have their packets captured by each sniffer. This election mechanism is executed when a sniffer captures for the first time a packet of a given node of the target network and takes into account the RSSI.

**Figure 3.**
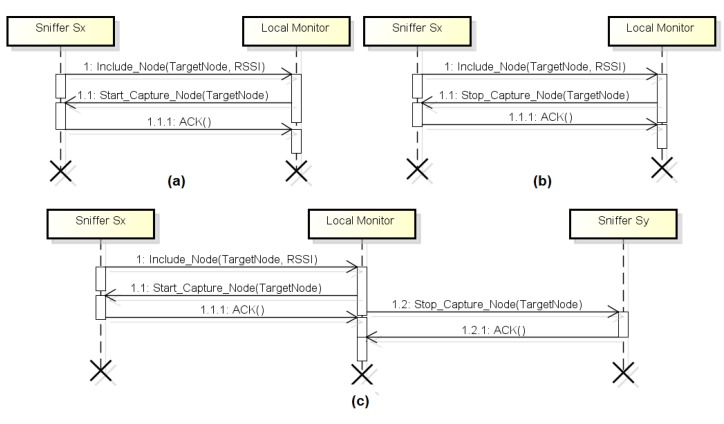
Sniffer election: (**a**) When no sniffer is capturing packets sent by the target node. (**b**) When S_Y_ is capturing packets sent by the target node and RSSI(S_Y_) ≥ RSSI(S_X_). (**c**) When S_Y_ is capturing packets sent by the target node and RSSI(S_Y_) < RSSI(S_X_).

As shown in the UML sequence diagram of Figure 3a, when a sniffer **S_X_** captures for the first time a packet of the target node **A**, it sends a message of inclusion of a new node to the local monitor reporting the address of this node **A** and the corresponding RSSI. If no other sniffer is capturing packets from node **A**, the local monitor sends a message to **S_X_** to start capturing the packets sent by node **A**. Thus, sniffer **S_X_** sends an ACK message to the local monitor and starts capturing packets sent by node **A**.

However, if another sniffer **S_Y_** is capturing packets from target node **A**, the local monitor analyzes which of the two sniffers is receiving packets from node **A** with the highest RSSI value, because, in general, the signal with the highest RSSI value has better quality. If **S_Y_** is receiving the signal from node **A** with the RSSI value greater than or equal to **S_X_**, the local monitor sends a message to **S_X_** stating that it should not capture packets sent by node **A**, as shown in Figure 3b. However, if **S_Y_** is receiving the signal from node **A** with the RSSI value less than **S_X_**, the local monitor sends a message to **S_X_** to start capturing packets from **A** and it sends a message to **S_Y_** to stop capturing packets from **A**, as shown in Figure 3c.

In all situations presented in sequence diagrams shown in Figure 3, after sending a message to a sniffer, the local monitor waits for an ACK message from this sniffer. If the ACK is not received after a time interval (timeout), the local monitor relays the message. If the local monitor does not receive any acknowledgment message after three transmission attempts, this sniffer is considered dead. In this case, the election mechanism is rebooted for the packets of the target nodes covered by this sniffer can be captured by another sniffer.

It is important to mention that RSSI captures the signal strength observed on the receiver antenna during packet reception. Two main considerations must be taken into account when analyzing RSSI. First, the RSSI calculation is solely based on correctly received packets, which implies that RSSI will not record packets that failed because of interference [24]. Second, the RSSI is not the average of the signal strengths measured through the reception of the whole packet. In fact, the RSSI value represents the received signal strength captured only during the reception of the preamble and header of the Physical Layer Protocol [25]. Thus, in cases where the interference affects only the data portion of the frame, this effect of interference will not be captured in the RSSI measurement [26]. Despite these considerations, the RSSI is a metric frequently used for evaluating the quality of the received signal.

To increase the RSSI precision, it has been suggested to use RSSI measurements together with statistical functions. For example, the RSSI value can be calculated from a fixed number of previous measurements or from the measurements in a time interval. Exponential Weighting Moving Average (EWMA) is also suitable to give more weight to recent measurements while not entirely discarding older ones. It is important to highlight that any type of statistical function can be seen as a stability mechanism able to significantly increase the representation of the specificities of wireless environments (e.g., reflections, absorptions, shadowing) [24].

Through the proposed election mechanism, in general only one sniffer captures packets sent by a given node of the target network, thereby reducing the transmission of packets captured by the monitoring network and thus reducing the energy consumption of this network. However, it is possible to capture redundant packets when the situation shown in Figure 3c occurs, due to the time interval between the messages 1.1 and 1.2. Redundant packets can also be captured due to the election mechanism to be run on the local monitor, because each local monitor assigns its monitoring nodes to target nodes, but different local monitors are not aware of each other's assignments. As shown in the results (Section 5), the proposed mechanism, although simple, considerably reduces the energy consumption of the monitoring network.

#### 3.1.2. Packet Capture

After running the sniffer election detailed in Section 3.1.1, sniffers start capturing packets, where each sniffer captures, in promiscuous mode, packets sent by the target nodes that it monitors, that is, nodes which were selected by this election mechanism.

**Figure 4.**
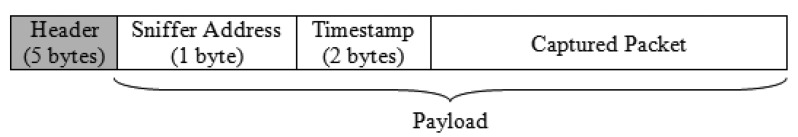
Format of the packet sent by sniffers without using aggregation of headers.

As mentioned in Section 3, the aggregation of headers is optional. When the aggregation of headers is not used, the sniffer generates, for each captured packet, a monitoring message containing its address (sniffer address), a timestamp and the bytes of the captured packet. This monitoring message is sent to the local monitor through the monitoring network using multihop routing. The format of the packet sent by the sniffer is shown in Figure 4. The header is inserted by the link layer protocol of the MicaZ platform and has fixed length of 05 bytes. The payload carries the monitoring message. The maximum payload size on the MicaZ platform is 29 bytes. Therefore, if the number of bytes of the monitoring message is larger than this value, the sniffer has to send more than one packet to the local monitor.

**Figure 5.**
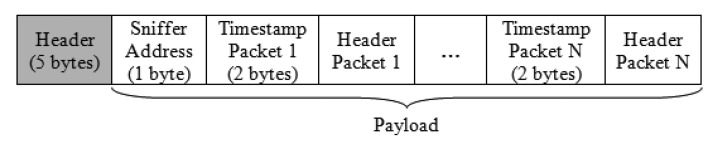
Format of the packet sent by sniffers using aggregation of headers.

When the aggregation of headers is used, only the information present in the headers of captured packets is sent by the sniffer to the local monitor. In this case, the headers of several packets may be sent in the same monitoring message, hence reducing the overhead of transmission and consequently reducing the energy consumption of the sniffers. The format of the packet sent by the sniffer is shown in Figure 5. The monitoring message carries the timestamp and the header of **N** captured packets. The maximum size of **N** depends on the size of header of the packets sent by the target nodes, because the monitoring message cannot be higher than the maximum payload size in the MicaZ platform (29 bytes).

### 3.2. Local Monitor

As described in Section 3.1.1, the local monitor runs the election mechanism together with sniffers. Moreover, the local monitor receives the monitoring messages sent by sniffers containing the packets captured from the target network and inserts the captured packets in a trace file (database) located on the server. For this purpose, the application running on the local monitor establishes a remote connection to a DBMS (DataBase Management System) installed on the server through an IP network. “MySQL Server 5.5” [27] is used in this work because it is a widely used DBMS. Besides, it is free software with GPL (General Public License).

As the captured packets are stored in a database, it is easy to get information about the target network using SQL (Structured Query Language) clauses. For example, the SQL clause “SELECT * FROM wsn WHERE source_addr = *10*” shows the packets sent by the target node whose address (*source_addr*) is 10.

The application running on the local monitor was implemented using the Java programming language because it is a multiplatform technology and enables the same code to run on different operating systems (Linux, Windows, *etc.*).

### 3.3. Server-Trace Analysis

The *trace analysis* application runs on the server (see Figure 1) and has the main function to extract information about the target network from the trace generated by local monitors. For this purpose, initially, the redundant packets that exist in the trace (database) are removed.

In the MicaZ platform, redundant packets can be detected by analyzing the Destination Sequence Number (DSN) present in the header of the packets sent by sensor nodes. The DSN has a size of 8 bits and it is incremented by the source node for each packet sent. When the DSN reaches the value of 255, the DSN of the next packet sent by the sensor node will have a value of 0 [28]. Therefore, if two or more packets have the same source address, the same DSN, and the difference between their timestamps is less than Δt, it means that they are the same packet. In this work, we assume Δt is equal to 10 s, because in our implementation a given sensor node does not send more than 255 packets in this time interval.

**Figure 6.**
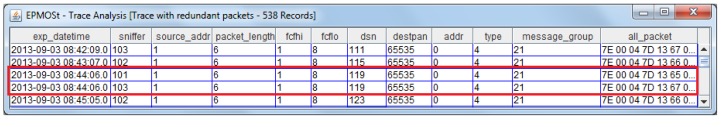
Example of trace with redundant packets.

Figure 6 shows a portion of the trace with the packets sent by the target node whose address (*source_addr*) is 1. As shown in Figure 6, there are two packets with DSN equal to 119 and they have the same timestamp (*exp_datetime*). In this case, one of these packets is removed from the trace.

**Figure 7.**
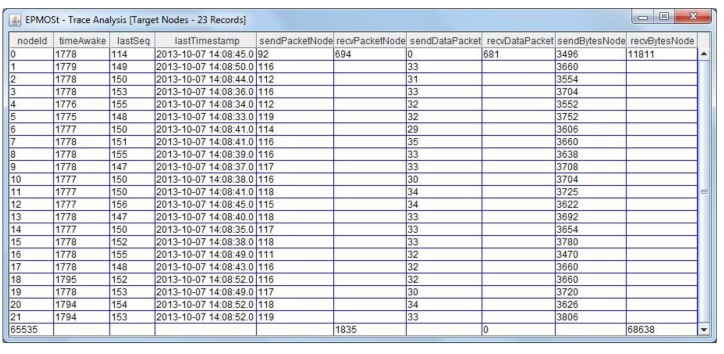
Example of information about the nodes of the target network.

After removing the redundant packets, the trace is analyzed to extract information about nodes and paths of the target network. Figure 7 illustrates an example of the *trace analysis* application. Note that it exemplifies information about the nodes of a given target network used to validate this application. Table 2 (Section 3.4.1) shows the description of each variable displayed in Figure 7.

In this case, as shown in Figure 7, only the node with addresses (*nodeId*) of **0** receives data packets, because it is the sink node. Nodes with addresses from **1** to **21** send data packets with information collected from sensors to the sink node. The address (*nodeId*) **65535** corresponds to the broadcast address of the network, where all packets of the routing protocol used by the target network are sent. The scenario used to obtain this information is described in Section 4.1.

**Figure 8.**
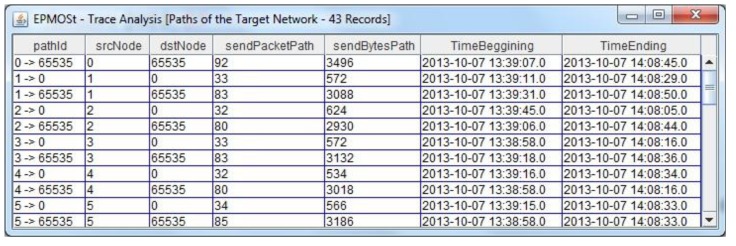
Example of information about the paths of the target network.

The screen of the *trace analysis* application shown in Figure 8 exemplifies information about some paths of the target network. Table 4 (Section 3.4.1) shows the description of each variable displayed in Figure 8. Note that each sensor node is the source of two paths, with one for the broadcast address (address 65535) and the other for the sink node (address 0). The *trace analysis* application was implemented using the Java programming language.

### 3.4. Server-SNMP Agent

As mentioned in Section 3, the information obtained from the analysis of the trace is stored in an MIB by an SNMP agent. The SNMP agent has been developed using the framework “*WebNMS SNMP Agent Toolkit Java Edition*” [29]. This framework enables rapid development of Java-based SNMP agents. Section 3.4.1 describes the proposed MIB for EPMOSt and Section 3.4.2 shows the tests performed to validate the operation of the SNMP agent developed in this work.

#### 3.4.1. MIB of the EPMOSt

An SNMP agent reads and writes management information in an MIB. The MIB is a data structure that stores managed objects whose values collectively reflect the current state of the managed devices. The values of these objects can be read and/or written by a management tool by sending SNMP messages to the agent. MIB objects are named hierarchically, so that any node in the tree can be identified by a sequence of names (or numbers) that specify the path from the root to the node.

**Figure 9.**
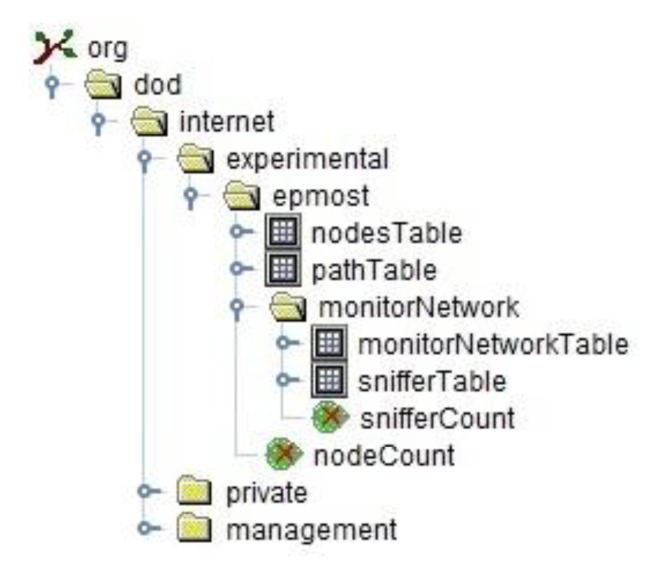
MIB of the EPMOSt.

The most widely used MIB is defined by RFC 1213 [30]. As shown in Figure 9, under the *Internet* node in this MIB are the subtrees *management*, *private* and *experimental*. Under the *management* node are the definitions of the MIB modules standardized by IETF (Internet Engineering Task Force). Under the *private* node are the definitions of objects of companies registered in the IETF. Under the *experimental* node are named objects that are being developed and tested. Therefore, the MIB of the EPMOSt has been defined under the *experimental* node.

The MIB of the EPMOSt has four tables: *nodesTable* that stores information about the nodes of the target network; *pathTable* that stores information about the paths of the target network; *monitorNetworkTable* stores statistical information about the monitoring network; and *snifferTable* for storing information about the sniffers. Also this MIB has two scalar objects: *nodeCount*, which is the number of nodes of the target network; and *snifferCount*, which is the number of sniffers.

Objects defined in each of the MIB tables are shown in Tables 2^3^, 4 and 5. Objects with an * beside their names have been identified from the study of passive monitoring systems for WSN, as discussed in Section 2 [6–8,10,11]. The identification of these objects is an additional contribution of this work. The remaining objects were captured in works that describe MIBs for WSN [11,31–33].

#### 3.4.2. Agent Validation

The SNMP agent developed in this work allows the integration of the monitoring system EPMOSt with any management tool with support for the SNMP protocol. In order to test and validate the agent, the management tools “*SNMP MIB Browser Android Tool*” [18] and “*Manage Engine MIB Browser Free Tool*” [19] were used. The main functionality of these tools is to provide communication with the agents, by sending SNMP messages to query and/or modify objects in an MIB.

**Figure 10.**
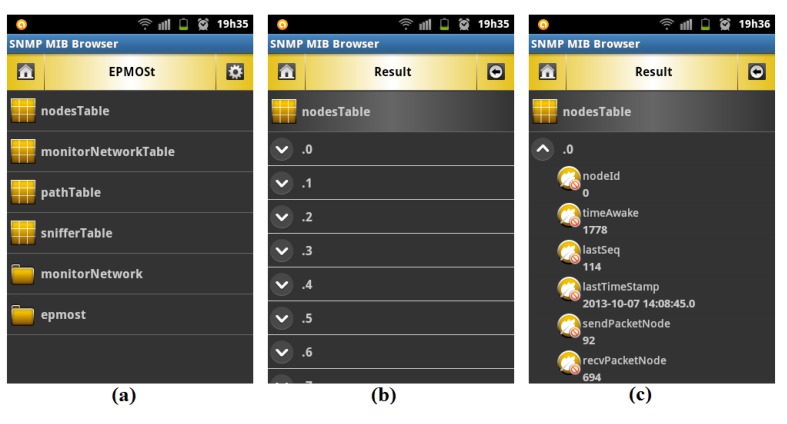
Operation of the “*SNMP MIB Browser Android Tool*”: (**a**) Viewing the MIB of the EPMOSt. (**b**) Viewing the target nodes. (**c**) Viewing information about the node **0**.

The “*SNMP MIB Browser Android Tool*” [18] was installed in a smartphone with Android operating system and queries were performed on all objects of the MIB of the EPMOSt to validate the operation of the agent developed in this work. All queries were performed successfully. Figure 10 exemplifies the operation of this tool. Figure 10a shows the structure of the MIB of the EPMOSt. By clicking *nodesTable* on the screen shown in Figure 10a, the user views the nodes of the target network (Figure 10b). By clicking on a given node in the screen shown in Figure 10b, the user views the information related to this node (Figure 10c).

The “*Manage Engine MIB Browser Free Tool*” [19] was also used to test and validate the SNMP agent. This software was installed on a computer with the Windows 7 operating system and queries were performed on all objects of the MIB of the EPMOSt. Again, all queries were performed successfully. Therefore, the tests performed with these two tools show that the agent developed in this work enables integration of EPMOSt with any management tool with support for the SNMP protocol.

## 4. Experiments

This section describes the experiments and the metrics used to evaluate the monitoring system EPMOSt.

### 4.1. Description

The experiments were performed by using MicaZ nodes with the TinyOS [23] operating system. The MicaZ platform has the following main features: microprocessor ATMEGA128L, 4KB of RAM memory, 128KB of ROM memory and a single radio frequency transceiver CC2420. The CC2420 radio uses the IEEE 802.15.4 standard and operates in an unlicensed frequency band of 2.4 GHz with a transmission rate of 250 Kbps [28].

**Figure 11.**
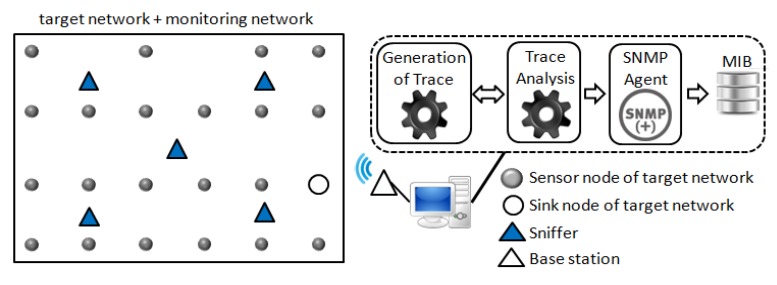
Scenario used in the experiments.

Figure 11 illustrates the scenario used for the experiments. The target network is composed of **21** sensor nodes and **01** sink node. The sensor nodes run an application that, every minute, measures the ambient temperature and sends the information to the sink node. The payload of the packets sent by the sensor nodes carries the measured temperature and a counter incremented at each temperature measurement.

**Figure 12.**
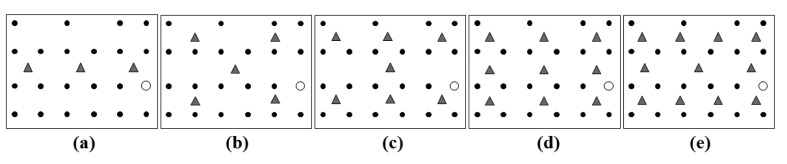
Distribution of sniffers: (**a**) 3 sniffers. (**b**) 5 sniffers. (**c**) 7 sniffers. (**d**) 9 sniffers. (**e**) 11 sniffers.

The monitoring network is composed of **N** sniffers and **01** base station. Sniffers capture the packets sent by the nodes of the target network and send these packets to the base station by using multihop routing. The base station sends the packets received from sniffers, through a USB cable, to a computer. In this scenario, the computer performs the functions of the local monitor (generation of the trace) and of the server, as shown in Figure 1. Experiments were performed with 3, 5, 7, 9 and 11 sniffers distributed in the monitored area, as shown in Figure 12.

For each scenario shown in Figure 12, three types of experiments were performed: “*election without aggregation*”, “*election with aggregation*” and “*without election*”. In the experiment “*election without aggregation*” sniffers run the application described in Section 3.1, in which the election mechanism proposed in Section 3.1.1 is implemented, but do not use the aggregation of headers proposed in Section 3.1.2. In the experiment “*elections with aggregation*”, sniffers run the election mechanism and the aggregation of headers.

In the experiment “*without election*” sniffers do not run any election mechanism and they capture all packets sent by the nodes of the target network that are within the coverage area of their radios. Thus, sniffers send all the bytes of captured packets to the local monitor, which then inserts these packets in the database. In this case, several sniffers can capture a single packet sent by a given node of the target network. It is noteworthy that this is the strategy used by all the monitoring systems described in Section 2 (SNTS, SNIF, Pimoto, LiveNet and PMSW).

### 4.2. Metrics

For the evaluation of the experiments the following metrics are defined: percentage of distinct packets captured by the monitoring network (% *Pcaptured*), energy consumed by the monitoring network for transmission of captured packets (*Et*) and average energy consumed by each sniffer for transmission of captured packets (*EtSniffer*).

In the EPMOSt, as well as all monitoring systems analyzed in Section 2, sniffers “listen” to all packets traveling in their radio interfaces. Therefore, the energy consumed in the reception of packets in the proposed system is similar to the energy consumed by the systems described in related works, and so it was not used as a metric for evaluation. However, if the duty cycle of the target network is known, each sniffer can be programmed to activate its radio interface just before the duty cycle start and to deactivate its radio interface just after the end of the duty cycle of the target network. This strategy contributes to further reduce the energy consumption of the monitoring network.

The total number of packets sent by the sensor nodes of the target network (*PsentTarget*) is obtained by Equation (1), where *CountInitial_i_* and *CountFinal_i_* are, respectively, the number of the first and last temperature measurements performed by node *i*, and *k* represents the number of sensor nodes of the target network. In the scenario used in these experiments, the value of *k* is 21:

(1) $$\text{PsentTarget}=\overset{k}{\underset{i=1}{\text{∑}}}\left({\text{CountInitial}}_{i}-{\text{CountFinal}}_{i}+1\right)$$

The number of distinct packets sent by node *i* captured by the monitoring network (*Pcaptured_i_*) is determined by checking which packets from node *i* are in the interval [*CountInitial_i_*, *CountFinal_i_*]. Therefore, the total number of distinct captured packets (*Pcaptured_i_*) is obtained by Equation (2):

(2) $$\text{Pcaptured}=\overset{k}{\underset{i=1}{\text{∑}}}{\text{Pcaptured}}_{i}$$

The percentage of distinct packets captured by the monitoring network (%*Pcaptured*) is determined by Equation (3):

(3) %Pcaptured=100∗Pcaptured/PsentTarget

As explained in Section 3.3, the number of redundant packets from node *I* (*Predundant_i_*) is determined by analyzing the DSN and the timestamp of the packets sent by the node. Therefore, the total number of redundant packets captured by the monitoring network (*Predundant*) is obtained by Equation (4):

(4) $$\text{Predundant}=\overset{k}{\underset{i=1}{\text{∑}}}{\text{Predundant}}_{i}$$

In order to calculate the energy consumed by the monitoring network for the transmission of packets, the energy model for MicaZ sensors defined in [34] and used in [20] was used. In this model, the energy consumed in the transmission (*Et*) is determined by Equation (5), in which *Psent* is the number of packets sent, *Plength* is the packet size in bytes, *TB* is the time spent for transmitting one byte, *It* is the value of current in the transmission mode and *V* is the electric voltage of the battery:

(5) Et=Psent×Plength×TB×It×V

The values used for *TB*, *It* and *V* were 32 mS, 17.4 mA and 3 volts, respectively. These values were obtained in the specification document of the MicaZ platform (datasheet), and are also equal to the values presented in [20,34]. By replacing these values in Equation (5), Equation (6) is obtained:

(6) $$Et=1,6704\times 10^{-6}\times \text{Psent}\times \text{Plength}$$

In the experiments “*without election*” and “*election without aggregation*”, the number of packets sent by the sniffers is determined by Equation (7). In these two types of experiments, each packet sent by the sniffers has size (*Plength*) of 23 bytes, with 05 bytes of header and 18 bytes of the monitoring message (see Figure 4). Therefore, the energy consumed by the monitoring network for transmitting the captured packets from the target network is determined by Equation (8):

(7) Psent=Pcaptured+Predundant

(8) $$Et=38,42\times 10^{-6}\times (\text{Pcaptured}+\text{Predundant})$$

When the aggregation of headers is used, as explained in Section 3.1.2, the headers of several packets of the target network are sent in the same monitoring message in order to reduce the energy consumption of the sniffers. In our experiments, the monitoring message carries the address of the sniffer (1 byte) and, the timestamp (2 bytes) and the header (11 bytes) of two packets of the target network. Therefore, in the experiment “*election with aggregation*” each packet sent by the sniffers has size (*Plength*) of 32 bytes, with 05 bytes of header and 27 bytes of the monitoring message (see Figure 5). In this experiment, the number of packets sent by the sniffers is determined by Equation (9). By replacing *Psent*, determined by Equation (9), and *Plength* equal to 32 in Equation (6), we obtain Equation (10), which corresponds to the energy consumed by the monitoring network for transmission of captured packets when the aggregation of headers is used:

(9) Psent=(Pcaptured+Predundant)/2

(10) $$Et=53,45\times 10^{-6}\times \left(\text{Pcaptured}+\text{Predundant}\right)/2$$

The average energy consumed by each sniffer for transmission of captured packets (*EtSniffer*) is determined by Equation (11), where *N* is the number of sniffers and *Et* may be calculated through Equation (8) or (10) according to the type of experiment:

(11) EtSniffer=Et/N

To exemplify how the variables used in Equations (1)–(11) affect the energy consumption, Table 6 shows the values of each variable for a single round with duration of 15 minutes for each type of experiment for a monitoring network with 11 sniffers (Figure 12e). It can be observed in Table 6 that the number of redundant packets captured by the monitoring network is zero when the election mechanism proposed in this paper is used. This occurs because in general only one sniffer captures packets sent by a given node of the target network, as explained in Section 3.1.1.

## 5. Results and Discussion

**Figure 13.**
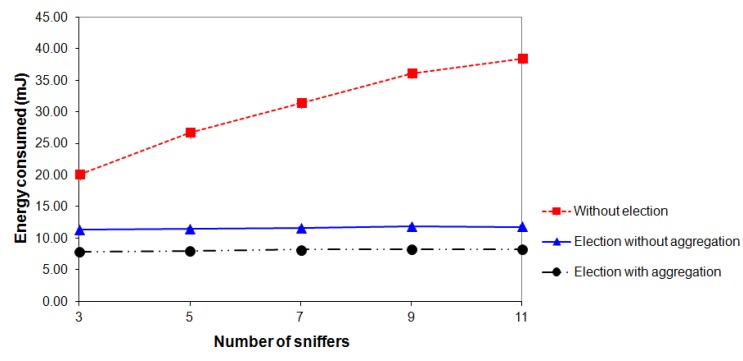
Energy consumed by the monitoring network *vs.* number of sniffers.

**Figure 14.**
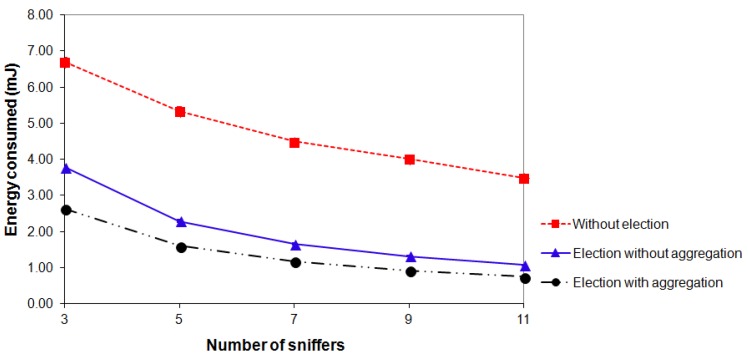
Energy consumed by each sniffer *vs.* number of sniffers.

**Figure 15.**
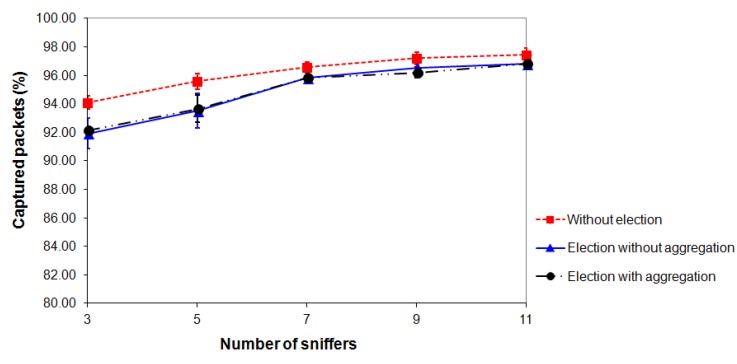
Packets captured by the monitoring network *vs*. number of sniffers.

For each of the scenarios shown in Figure 12 (Section 4.1), three types of experiments were performed: “*election without aggregation*”, “*election with aggregation*” and “*without election*”. For each scenario and for each type of experiment, 10 rounds with duration of 15 min were performed. All experiments were conducted in the same environment under the same conditions. The results shown in Figures 13, 14 and 15 refer to the average values of 10 rounds with a confidence interval of 95%. The margins of error of the experiments can be perceived only in Figure 15 because, in Figures 13 and 14, their values are very small and cannot be viewed.

We first investigate the energy consumed by the monitoring network for transmitting the captured packets as a function of the number of sniffers. As shown in Figure 13, when the election mechanism is not used, the energy consumed by the monitoring network increases when the number of sniffers increases. This occurs because the packets sent by a given node of the target network are captured by a larger number of sniffers, thereby increasing the number of redundant captured packets and consequently increasing the energy consumed by the monitoring network for the transmission of these packets. In the two types of experiments in which the election mechanism is used, the energy consumption of the monitoring network remains almost constant, because, when the number of sniffers increases, each sniffer captures packets from a smaller number of nodes of the target network, but the total number of packets sent by the monitoring network changes very little.

Figure 13 shows that, with 11 sniffers, the energy consumed by the monitoring network is 38.4 mJ when the election mechanism and the aggregation of headers are not used. When the election mechanism without aggregation of headers is used, the energy consumption is 11.8 mJ, which corresponds to a decrease of 69.3%. This decrease in energy consumption occurs because the use of the election mechanism significantly reduces the number of redundant packets transmitted by the sniffers. When the election mechanism and the aggregation of headers are used, the energy consumption is only 8.27 mJ, which corresponds to a decrease of 78.5%. These results prove the energy efficiency of the EPMOSt.

Next, we study the average energy consumed by each sniffer for transmitting the captured packets as a function of the number of sniffers. Figure 14 shows that, for all types of experiments, a decrease in the energy consumed by each sniffer when the number of sniffers is increased, occurs. This is because sniffers constitute an independent mesh network. Thus, when the number of sniffers increases, sniffers are closer to each other and the power of their radio interfaces is reduced, thereby reducing the coverage area of each sniffer and, consequently, each sniffer captures packets from a smaller number of target nodes. It can also be seen that the use of the election mechanism considerably reduces the energy consumption of the sniffers and consequently prolongs the lifetime of the monitoring network.

Finally, we study the percentage of distinct packets captured by the monitoring network as a function of the number of sniffers. Figure 15 shows that, for all types of experiments, when the number of sniffers increases, the percentage of captured packets also increases. This occurs because the sniffers are closer to the nodes of the target network and they receive the radio signals with higher strength (RSSI). When the election mechanism is not used, the percentage of captured packets is slightly higher than in the two types of experiments that use the election mechanism. This occurs because the same packet may be captured by more than one sniffer, thereby increasing the probability of it being captured. However, this difference between the captured packets decreases with increasing number of sniffers and it is only 0.62% at 11 sniffers.

It is important to emphasize that the sniffers used in these experiments have only one radio interface and, consequently, they cannot receive and send packets at the same time. This contributes to the monitoring network to capture less than 100% of packets sent by the target network. Therefore, the results should be better if the sniffers had two radio interfaces.

The results presented in this section demonstrate that EPMOSt considerably reduces the energy consumption of the monitoring network, when it is compared with the monitoring systems described in Section 2 (which do not use the election mechanism of sniffers). Besides, EPMOSt keeps the percentage of captured packets close to the values obtained without the use of the election mechanism. These results prove the viability of using EPMOSt when there is a need of monitoring a WSN in a real scenario over long periods.

## 6. Conclusions

In this work, initially, we have analyzed and compared five passive monitoring systems proposed for WSN: SNTS, SNIF, Pimoto, LiveNet and PMSW. However, none of these systems aims to reduce the energy consumption of the monitoring network. Within this context, in this work we propose an energy-efficient passive monitoring system for WSN named EPMOSt that reduces the energy consumption of the monitoring network and provides monitoring information using an SNMP agent.

All modules of the EPMOSt have been described and validated. Experiments were performed using the MicaZ platform and their results show that the election mechanism used in our system reduces, by up to 69.3% (11 sniffers), the energy consumed by the sniffers for transmitting captured packets. When the election mechanism and the aggregation of headers are used, the energy consumption decreases by 78.5% (11 sniffers). However, although the proposed election mechanism to achieve a rate of captured packets is slightly smaller, this rate increases with the number of sniffers and is reduced by only 0.62% when the monitoring network has 11 sniffers. Therefore, the results of the experiments demonstrate the viability of using the EPMOSt to monitor WSN in real scenarios, because the reduction of energy consumption of the monitoring network contributes to prolong the lifetime of this network. It was also shown that the SNMP agent developed in this work enables the integration of EPMOSt with any management tool which supports the SNMP protocol, facilitating the management of the target network.

The contributions presented in this work bring up interesting perspectives for future research. We highlight three main directions. Firstly, we intend to change the election mechanism to take into account RSSI, LQI (Link Quality Indicator), level of battery of the sniffers, and the number of target nodes monitored by each sniffer, aiming to balance the energy consumption of the sniffers and prevent some sniffers from having their energy depleted long before others. Secondly, we intend to conduct new experiments to evaluate the lifetimes of the monitoring network and the target network. Finally, we will use a network simulator to simulate the operation of the EPMOSt in a network with higher density in order to evaluate its scalability.

## Figures and Tables

**Table 1.** Characteristics of monitoring systems.

**Characteristic**	**SNTS [6]**	**SNIF [7]**	**Pimoto [8]**	**LiveNet [10]**	**PMSW [11]**
Energy-efficient	No	No	No	No	No
Analysis mode	Offline	Online	Online	Offline	Online
Captured packets	Data + Control	Data + Control	Data + Control	Data	Data + ACK
Event analysis	Faults analysis	Faults analysis	None	None	Faults analysis and performance assessment
Redundant packets	Yes	Yes	No	Yes	Yes
Aggregation	No	No	No	No	No
Visualization tool	Developed by the authors	Developed by the authors	Wireshark plugin	Developed by the authors	Developed by the authors

**Table 2.** Objects represented in the *nodesTable*.

**Object Name**	**Object Description**
*nodeId*	Address of the target node
*timeAwake*	Time (seconds) at which the node is active
*lastSeq*	DSN of the last packet sent by the node
*lastTimestamp*	Timestamp of the last packet sent by the node
*sendPacketNode*	Number of packets sent by the node
*recvPacketNode*	Number of packets received by the node
*sendDataPacket* *	Number of data packets sent by the node
*recvDataPacket* *	Number of data packets received by the node
*sendBytesNode*	Number of bytes sent by the node
*recvBytesNode*	Number of bytes received by the node

**Table 3.** Objects represented in the *snifferTable*.

**Object Name**	**Object Description**
*snifferId*	Address of the Sniffer
*lastTimestampSniffer*	Timestamp of the last packet captured by the sniffer
*capturedPacket*	Number of packets captured by the sniffer
*capturedBytes*	Number of bytes captured by the sniffer

**Table 4.** Objects represented in the path*Table*.

**Object Name**	**Object Description**
*pathId*	*Path* in format source → destination
*srcNode*	Address of the source node of the path
*dstNode*	Address of the destination node of the path
*sendPacketPath*	Number of packets sent through the path
*sendBytesPath*	Number of bytes sent through the path
*timeBeginning*	Timestamp of the first packet sent through the path
*timeEnding*	Timestamp of the last packet sent through the path

**Table 5.** Objects represented in the *monitorNetworkTable*.

**Object Name**	**Object Description**
*targetNode*	Address of the target node
*packetSentTarget*	Number of packets sent by the target node
*packetCaptured*	Number of packets captured from the target node
*distinctPacketCaptured* *	Number of distinct packets captured from the target node
*redundantPacketCaptured* *	Number of redundant packets captured from the target node
*packetNotCaptured* *	Number of packets not captured from the target node

**Table 6.** Values of variables for a single round with 11 sniffers.

**Type of Experiment**			
**Variable**	**without Election**	**Election without Aggregation**	**Election with Aggregation**
*PsentTarget*	325	324	324
*Pcaptured*	316	312	316
*%Pcaptured*	97.23%	96.30%	97.53%
*Predundant*	679	0	0
*Et* (mJ)	38.23	11.99	8.45
*EtSniffer* (mJ)	3.48	1.09	0.77

## References

[1] Borges N.J.B., Ribeiro N.P.F., Andrade R.M.C. (2010). Wireless Sensor Networks Advances for Ubiquitous Computing. Designing Solutions-Based Ubiquitous and Pervasive Computing; New Issues and Trends.

[2] Ringwald M., Romer K. Deployment of sensor networks: Problems and passive Inspection.

[3] Ramanathan N., Chang K., Kapur R., Girod L., Kohler E., Estrin D. Sympathy for the Sensor Network Debugger.

[4] Hanninen M., Suhonen J., Hamalainen T.D., Hannikainen M. Practical monitoring and analysis tool for WSN testing.

[5] Liu Y., Liu K., Li M. (2010). Passive Diagnosis for Wireless Sensor Networks. IEEE/ACM Trans. Netw..

[6] Khan M.M.H., Luo L., Huang C., Abdelzaher T. SNTS: Sensor network troubleshooting suite.

[7] Ringwald M., Romer K. Deployment of sensor networks: Problems and passive Inspection.

[8] Awad A., Nebel R., German R., Dressler F. On the need for passive monitoring in sensor networks.

[9] Wireshark http://www.wireshark.org.

[10] Chen B.R., Peterson G., Mainland G., Welsh M. LiveNet: Using passive monitoring to reconstruct sensor network dynamics. Proceedings of the IEEE.

[11] Xu X., Wan J., Zhang W., Tong C., Wu C. (2011). PMSW: A passive monitoring system in wireless sensor networks. Int. J. Netw. Manag. IJNM.

[12] Sommer P., Kusy B. Minerva: Distributed tracing and debugging in wireless sensor networks.

[13] Jiangwu N., Huadong M., Lufeng M. Passive Diagnosis for WSNs Using Data Traces.

[14] Yang Y., Xu Y., Li X. (2011). Lossy nodes inference based on end-to-end passive monitoring in wireless sensor networks. High Technol. Lett..

[15] Romer K., Ringwald M. Increasing the visibility of sensor networks with passive distributed assertions.

[16] Nagios http://www.nagios.org.

[17] Net-SNMP http://www.net-snmp.org.

[18] SNMP MIB Browser Android Tool. http://www.manageengine.com/free-snmp-mibbrowser-android.

[19] Manage Engine MIB Browser Free Tool. http://www.manageengine.com/products/mibbrowser-free-tool.

[20] Rocha A.R., Pirmez L., Delicato F.C., Lemos E., Santos I., Gomes D.G., Souza J.N. (2012). WSNs Clustering Based on Semantic Neighborhood Relationships. Elsevier Comput. Netw..

[21] Garcia F.P., Souza J.N., Andrade R.M.C. An energy-efficient passive monitoring system for wireless sensor networks.

[22] Gay D., Levis P., Behren R.V., Welsh M., Brewer E., Culler D. The NesC Language: A holistic Approach to Networked Embedded System.

[23] Hill J., Szewczyk R., Woo A., Hollar S., Culler D., Pister K. System Architecture Directions for Networked Sensors.

[24] Borges V.C.M., Curado M., Monteiro E. (2011). Cross-layer routing metrics for Mesh networks: Current status and research directions. Comput. Commun..

[25] IEEE IEEE Standard for Information Technology—Telecommunications and Information Exchange Between Systems—Local and Metropolitan Area Networks—Specific Requirements—Part 11: Wireless LAN Medium Access Control and Physical Layer Specifications; IEEE 2007. http://ieeexplore.ieee.org/xpl/articleDetails.jsp?arnumber=STDPD97235&-contentType=Standards.

[26] Vlavianos A., Law L.K., Broustis I., Krishnamurthy S.V., Faloutsos M. Assessing Link Quality in IEEE 802.11 Wireless Networks: Which is the Right Metric?.

[27] MySQL http://mysql.org.

[28] MPR-MIB Users Manual—Crossbow Technology http://www-db.ics.uci.edu//pages/research/quasar/MPR-MIB%20Series%20User%20Manual%207430-0021-06_A.pdf.

[29] WebNMS SNMP Agent Toolkit Java Edition http://www.webnms.com/javaagent.

[30] McCloghrie K., Rose M. (1991). Management Information Base for Network Management of TCP/IP-Based Internets: MIB-II. RFC 1213. http://www.ietf.org/rfc/rfc1213.txt.

[31] Jacquot A., Chanet J., Mean H.K., Diao X., Li J.-J. LiveNCM: A new wireless management tool.

[32] Zhang B., Li G. Survey of network management protocols in wireless sensor network.

[33] Ye J., Zhao Z., Li H., Chen H. Hierarchical heterogeneous wireless sensor network management system.

[34] Jurdak R., Ruzzelli A.G., O'Hare G. Adaptive radio modes in sensor networks: How deep to sleep?.

